# How Do Intellectual Property Law and International Trade Agreements Affect Access to Antiretroviral Therapy?

**DOI:** 10.1371/journal.pmed.0030332

**Published:** 2006-08-08

**Authors:** Michael Westerhaus, Arachu Castro

## Abstract

Westerhaus and Castro argue that US-negotiated bilateral, regional, and multilateral trade agreements are hindering access to essential HIV medicines in poor countries.

In recent years, globalization has forced a deeper appreciation of the relationship between intellectual property (IP) law and global health. The threat of an emergent avian bird flu pandemic led to calls for Roche to relax patent restrictions on oseltamivir, a drug with potential efficacy against bird influenza [1,2]. In the context of the fall 2001 anthrax attacks, the US government faced pressure to break Bayer's patent on ciprofloxacin in order to increase availability of the drug [3]. Such situations have generated intense debate over the value of patent protection amidst health crises.

Nowhere have these debates been more intense than around the issue of global access to HIV treatment. Multinational pharmaceutical companies, World Trade Organization (WTO) members, US and European Union trade representatives, and health-care activists have clashed over provision of antiretroviral therapy (ART) to people living with AIDS in developing countries. The debate centers upon the value and role of patents obtained for HIV-related pharmaceutical products, drug-manufacturing techniques, and forms of drug delivery. These arguments have recently intensified amidst an increased US pursuit of bilateral, regional, and multilateral trade agreements—which include strong IP provisions—with low- and middle-income countries throughout the world.

In this paper, we examine the key areas of concern regarding access to ART related to US-negotiated bilateral, regional, and multilateral trade agreements. We first examine developments in IP law in the wake of WTO's Doha Declaration, which affirmed the priority of public health over the protection of patents. We look specifically at those developments with particular salience for health-related issues and link this history with the current context of access to antiretrovirals (ARVs) worldwide. Next we map out the key claims about, and questions surrounding, the role of patent law, followed by a critical look at the impact of trade agreements on IP law and their potential threat to global health. Finally, we suggest policy and advocacy strategies to ensure and promote access to ART in the era of US-led attempts to strengthen global IP law through the vehicle of “free” trade agreements.

## IP Law as It Relates to Public Health: Doha and Beyond

The Trade-Related Aspects of Intellectual Property Rights (TRIPS) agreement, completed in 1994, revolutionized global patent law by requiring the standardization of IP law among all WTO members by January 1, 2005. Immediately, concern arose that the TRIPS agreement would constrain the protection of public health in low- and middle-income countries. In response, WTO delegates gathered in Doha, Qatar, on November 14, 2001, issued a strong statement, now referred to as the Doha Declaration [4].

Delegates agreed at Doha that the least-developed country members (defined as such by the United Nations based on a series of indicators including income, nutrition, health, education, literacy, and economic vulnerability [5]) were not obliged to implement patent law for pharmaceuticals until January 1, 2016. Of the 50 least-developed countries, 32 are WTO members.

Additionally, the Doha Declaration acknowledged the short-sightedness of the TRIPS agreement rule mandating that countries could break patents only in public health emergencies in order to produce generic drugs “predominantly for the supply of the domestic market”[6]. This wording left countries without domestic production capacity unable to access generic medications for their populations. In paragraph 6 of the Doha Declaration, WTO officials ordered the TRIPS council to develop a plan to address this problem by the end of 2002: “We recognize that WTO members with insufficient or no manufacturing capacities in the pharmaceutical sector could face difficulties in making effective use of compulsory licensing [the granting of a license to another producer to manufacture, use, and distribute generic versions of patented inventions without the consent of the patent holder but in exchange for a remuneration to compensate for the reduction of the potential market for the branded sale of the drugs] under the TRIPS Agreement. We instruct the Council for TRIPS to find an expeditious solution to this problem and to report to the General Council before the end of 2002” [4].

Reaching consensus on what became known as the “paragraph 6 problem” created great debate among TRIPS council members. The US led an effort to restrict the provisions of paragraph 6 of the Doha Declaration to certain diseases: namely, AIDS, malaria, tuberculosis, and other infectious diseases creating epidemics. In addition to trying to limit the use of compulsory licenses, the US worked to limit the number of countries that could benefit from the importation of generic medications [7]. On August 30, 2003, the TRIPS council finally issued the “Decision on Implementation of Paragraph 6 of the Doha Declaration,” stating that countries without manufacturing capacities—a definition that still remains unclear—could declare compulsory licenses and on that basis alone legally import generic medications. However, this decision was only a temporary waiver until a permanent amendment could be agreed upon.

Efforts to agree upon a permanent amendment to TRIPS were fraught with further discord. The US and other developed countries argued for ratification of the temporary waiver as a permanent amendment. On the other hand, developing countries, led by the African Group, argued that the temporary waiver included too many procedural obstacles that would still hinder access to essential medications for countries without domestic production capacity [8]. Médecins Sans Frontières pointed out that no country had actually used the temporary amendment and argued that it would be unwise to make permanent something that had not been tested [9].

Despite these concerns, WTO members agreed in early December 2005, just prior to the WTO Ministerial Conference in Hong Kong, to make the temporary waiver permanent if at least two-thirds of the 148 WTO members ratified the amendment by December 1, 2007 [10]. The US and the Pharmaceutical Research and Manufacturers of America (PhRMA) touted the amendment as “part of the wider national and international action, including many activities taken by PhRMA companies, to address the gravity of the public health problems afflicting many developing and least-developed countries” [11]. Yet do these acts of apparent generosity translate into tangible advances in access to essential medicines in low- and middle-income countries?

## Have WTO Rules Improved Access to ART?

For all the wrangling over the specific provisions of the TRIPS agreement and the self-proclaimed interest by multinational pharmaceutical companies and the US government in promoting global health, we argue that little has changed to suggest that WTO rules improve global public health. In fact, compulsory licenses, the primary mechanism offered for public health protection by the TRIPS agreement and the Doha Declaration, have rarely been used [12]. The exact procedures for issuing a compulsory license for ARV production remain unclear and largely untested. Significant international pressure also exists against declaring compulsory licenses—as seen when Brazil recently threatened to issue compulsory licenses for efavirenz, lopinavir/ritonavir, and tenofovir [13,14]. For these reasons in part, only four countries—Malaysia, Indonesia, Zambia, and Mozambique—have thus far issued compulsory licenses for ARV production, all of them in 2004 [15].

No country has yet made use of the provisions instilled in the temporary waiver, even though many low- and middle-income countries face public health emergencies. Southern Africa, for example, is currently witnessing a decimation of its population by AIDS, while malaria kills at least 1 million people, predominantly children, per year. So why aren't these countries using the waiver provisions? Some might suggest the old mantra that low- and middle-income governments are too corrupt and power-seeking to actually care about the health of their populations. But if this were true, how could we explain the health successes of countries such as Brazil, Cuba, and Thailand? An alternative explanation, as suggested by the African Group, is that WTO rules are far too cumbersome and impractical for poor countries to navigate. Viewed in this light, the humanitarian motives pled by pharmaceutical companies and economically powerful governments can be seen as empty lip service, functioning only to counter growing calls for social justice in global health.

Pharmaceutical companies also argue that patents are central to the preservation of innovation. Yet there is little evidence that current IP law creates incentives for the development of new drugs. An analysis of a small sample of pharmaceutical inventive activity before and after compulsory licensing showed no uniform decline in scientific innovation [16], challenging the assumption that patent protection is necessary to foster the development of new drugs. Furthermore, current patent protections do not necessarily create financial incentives for the development of desperately needed drugs, such as a malaria vaccine, in poor countries: between 1975 and 1997, only 13 out of 1,223 new drugs introduced globally were specifically targeted toward diseases disproportionately affecting poor countries [17].

In support of the research-based pharmaceutical companies that produce brand-name drugs, some argue that patent laws have historically played very little role in inhibiting access to essential medicines in the developing world, asserting instead that poverty and poor health infrastructure are the primary obstacles to ARV distribution [18,19]. Additionally, poor drug quality, inadequate public health infrastructure, understaffed clinics and hospitals, lack of political commitment, and underfinancing of HIV treatment programs are cited as major factors obstructing the provision of ART. While recognizing the ability of these factors to impede access to ARVs, and working for their elimination, health activists counter that patent law—because of its role in determining drug prices—also creates a formidable barrier to access to medicines.

Health activists and academics argue that current patent protection, by eliminating competition, generally leads to higher prices [20,21], which directly obstructs the promotion of global health equity. Due to the enormity of the AIDS pandemic, health activism has recently focused upon HIV treatment, charging that current IP law impedes the purchase of ARVs in resource-poor settings and allows pharmaceutical companies to monopolize the markets of developing nations [22–25]. As a result, the cost of ARVs far exceeds personal and national budgets, and the development of more affordable generic alternatives is proscribed. This cost not only represents an insurmountable barrier to initiating treatment but also could hamper complete adherence and trigger drug resistance for those already on therapy [26]. From the vantage point of health activists, alternatives to current patent law and incentive mechanisms, such as regulatory flexibility (which could allow the mix of local production and imports of generic drugs) [21,27] or “pull” programs to stimulate research for vaccine development [28], are crucial to alleviate the suffering of people living with treatable diseases in poor countries.

## Access to ARVs and Patent Law

Increasing ART distribution is a public health imperative. Currently, 40.3 million people in the world live with HIV. In 2005, 3.1 million people in the world died of AIDS; of these, about 570,000 were children [29]. HIV medications dramatically decrease HIV mortality and morbidity, and, when ARVs are made available, HIV treatment in resource-poor settings has been shown to be extremely successful [30].

Between December 2003 and 2005, the World Health Organization (WHO) led an effort, called the “3 by 5” initiative, to rapidly scale up HIV treatment worldwide, aiming to treat 3 million people with ART by the end of 2005. During the initiative, the number of patients receiving ART in low- and middle-income countries increased from 400,000 to 1.3 million [31]. Although short of the December 2005 goal, the initiative achieved significant progress in mobilizing the expansion of ART. WHO estimates that between 250,000 and 350,000 premature deaths were averted due to the scale-up in ART. Despite these successes, many challenges remain in improving access to ART. Some of the reasons cited by WHO to explain the failure to reach the 3 by 5 initiative's targets include poorly harmonized partnerships; constraints on the procurement and supply of drugs, diagnostics, and other commodities; strained human resources capacity and other critical weaknesses in health systems; and difficulties in ensuring equitable access [31].

In addition to these challenges, global changes in IP law threaten to slow down or even reverse gains made in improving access to ART. Most types of ARVs, of which 12 are included in the WHO list of essential medicines [32], are produced by generic manufacturers in India [33], where it is estimated that 5.1 million adults and children are living with HIV [34]. Indian generic companies such as Cipla and Ranbaxy, capitalizing on the country's substantial economic and infrastructural capabilities for drug production, have become the major suppliers of low-cost ART regimens throughout the developing world [35]. Médecins Sans Frontières estimates that 50 percent of these medications are produced in India [36]. However, this supply of inexpensive generic ARVs may soon end as a result of India's obligation to enforce patent protection for medicines since January 1, 2005, in accordance with stipulations laid out in the 1996 TRIPS agreement—changes that have caused great concern among those working to expand HIV treatment. Believing that strengthened patent law would increase opportunities for foreign investment within India, the Indian government passed amendments in March 2005 that boosted IP law and could hinder the future production of medications for health emergencies such as AIDS [37–39].

These developments have generated worldwide concern that access to affordable ART, especially for second- and third-line ARVs, may be severely constrained under India's enforcement of TRIPS. In a December 17, 2004, letter to the Indian Minister of Health, Jim Y. Kim, then director of the Department of HIV/AIDS at the WHO cautioned India against implementing new patent law that would hinder public health efforts both within and outside of India [40]. Indian health ctivists declared that “the Government is adopting a simplistic, conformist approach of hurriedly ‘aligning’ our Patent Law to the coercive version of TRIPS” and asserted that “the need of the hour is to follow a more creative nd independent approach, while still remaining within the broad contours of TRIPS” [41]. Many worry that the patent law changes in India will end the Indian supply of cheap, generic ART, thereby resulting in higher medication prices and the imposition of permanent obstacles that may unnecessarily thwart efforts to deliver ART to the poor. This issue of access to medicines is at the heart of the current and historical controversy over the role of IP law. The issue forces us to confront the question of whether profit or human health should take priority.

## US Trade Policy and Access to ARVs

In January 2003, President Bush announced his five-year plan that would allocate $15 billion to global programs aimed at HIV treatment and prevention, now referred to as the President's Emergency Plan for AIDS Relief (PEPFAR) [42]. Working in 15 focus countries, this initiative endeavors to support treatment for 2 million people living with HIV/AIDS, to prevent 7 million new infections, and to support care for 10 million people infected with or affected by HIV/AIDS by 2008.

After just eight months of operation, PEPFAR reported rapid progress in achieving its aims—by March 2005, 155,000 people were receiving ART, 1.2 million women and infants had benefited from measures to prevent mother-to-child transmission of HIV, and 1.7 million individuals infected with or affected by HIV/AIDS were receiving supportive care under its auspices [43]. Moreover, at the 2005 Summit of the Group of Eight Nations (G8), the heads of state of the eight wealthiest countries pledged additional aid with a focus on AIDS in Africa, and surmised that universal access to HIV treatment could be possible by 2010 [44].

However, recent US trade policy threatens to undermine these advances in improving access to ARVs. After failing to promote “free” trade on hemispheric and global levels, the US has embarked on an aggressive campaign to liberalize trade through bilateral, regional, and multilateral trade agreements (Box 1). These agreements have conditioned liberalized trade upon the expansion of IP law for multinational pharmaceutical companies holding patents for ARVs, among other essential medicines. Specifically, these agreements extend the protection of patents beyond the 20-year period (Box 2), freeze generic manufacturing of ARVs, protect the manufacturers' drug testing data for five years (a practice known as data exclusivity), and limit options for compulsory licensing. Additional measures include a reduction in the number of inventions, such as “diagnostic, therapeutic, and surgical methods,” that can be excluded from patent law, the allowance of known substances to be patented again for each new use, and provisions requiring national drug regulatory authorities to block registration of generic medications. Such broadened IP rules beyond those negotiated in the WTO TRIPS agreement are now referred to as “TRIPS-plus” measures [45].

**Box 1. Trade Liberalization around the World**

- US Bilateral Trade Agreements: Agreements negotiated between the US and one other country. Examples include the US–Chile Free Trade Agreement (FTA), the US–Peru FTA, the US–Colombia FTA, and the US–Australia FTA.
- US Regional Trade Agreements: Agreements negotiated between the US and a number of neighboring countries. Examples include the North American Free Trade Agreement (NAFTA), the US–CAFTA, and the US–South African Customs Union Free Trade Negotiations.
- Multilateral Trade Agreements: Agreements negotiated at a global level. The TRIPS agreement is an example.

**Box 2. Timeline of Key Events Related to Pharmaceutical IP Law**

1883: Paris Convention establishes 20-year patent protection.

1986–1994: Uruguay round of trade negotiations occurs, during which the TRIPS agreement is formulated.

1995: The WTO is established, and the TRIPS agreement is implemented.

1999: Large-scale protests against globalization occur during the WTO Ministerial Conference in Seattle.

2001 November: Doha Declaration is made in Qatar.

2003 August: Temporary waiver is established to address the “paragraph 6 problem.”

2004 May: US and Central American countries sign CAFTA with TRIPS-plus measures.

2005 March: India passes amendments to become TRIPS-compliant.

2005 December: US completes trade agreement with Peru with TRIPS-plus measures.

2006 February: US completes trade agreement with Columbia with TRIPS-plus measures.

“TRIPS-plus” measures are included in agreements recently signed and in others currently being negotiated. For example, in May 2004, the US signed the Central American Free Trade Agreement (CAFTA) with Costa Rica, El Salvador, Guatemala, Honduras, and Nicaragua (the Dominican Republic was added in August 2004). CAFTA requires both data exclusivity for five years and patent extensions to offset delays in the granting of a patent [46]. Additionally, the US has signed similar bilateral trade agreements with Singapore, Chile, Peru, Colombia, and Morocco, and is currently working on bilateral agreements with Panama and Thailand and a regional agreement with the Southern African Custom Union (Botswana, Lesotho, Namibia, South Africa, and Swaziland).

Health activists, academics, developing country governments and clinicians working in resource-poor settings are concerned that these agreements will greatly augment the power of research-based pharmaceutical companies that produce brand-name drugs in the markets of developing nations, thereby greatly compromising access to ARVs. The extension of patent law beyond the provisions delineated in the TRIPS agreement is worrying. Trade agreements currently being negotiated may severely constrain production of generic drugs, the primary source of affordable medications in resource-poor settings. TRIPS-plus provisions continue a tradition of limiting access to ART for the poor by instituting measures that condone high drug prices. As a reaction to these limitations, in May 2006 ten South American countries issued a joint declaration on IP committing themselves “to avoid TRIPS plus provisions in bilateral and regional trade agreements,” among other similar measures [47].

In addition to the uneasiness expressed by activists, clinicians, and researchers, similar concerns have recently been voiced from within the US government. On September 30, 2004, 12 members of the US House of Representatives submitted a letter to President Bush expressing opposition to the IP provisions in CAFTA and other “free” trade agreement negotiations with the Andean countries and Panama. Authors of the letter criticized the lack of specific language on the right to compulsory licensing and parallel importation and the imposition of five-year blockades on drug testing data. They warned that these agreements could violate the TRIPS agreement and the Doha Declaration [48].

Further, TRIPS-plus measures may have harmful consequences for PEPFAR, a program ostensibly predicated on a vision for improved global health. Professing a desire to ensure that the poor receive the best HIV treatment medications possible, PEPFAR uses a stringent system for determining which drugs may be used in their treatment program. Originally, only brand-name ARVs were used in the start-up phases of PEPFAR. However, criticism about the exorbitant costs associated with brand-name drugs forced PEPFAR to consider using cheaper generic medications, thereby allowing for increased HIV treatment. Generic ARVs—such as lamivudine, zidovudine, and nevirapine—produced by companies in South Africa and India received FDA approval in 2005 with the hope that this would allow greater numbers of patients to be treated [49–51]. However, strengthened IP provisions, such as TRIPS-plus measures, threaten to prevent future production of low-cost, generic ARV alternatives for use in PEPFAR. The pursuit of TRIPS-plus measures stands counter to the lofty aims to address global health through initiatives such as PEPFAR (Figure 1).

**Figure 1:**
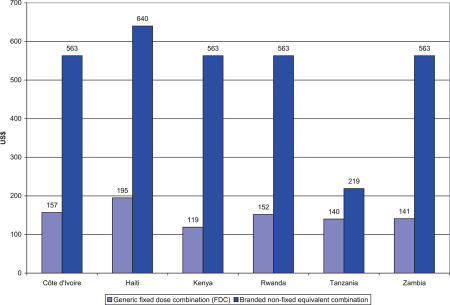
Annual Treatment Cost of Stavudine 40 mg + Lamivudine 150 mg + Nevirapine 200 mg per Patient in Countries Receiving PEPFAR Funding for which Cost Information Is Available Source: Global Price Reporting Mechanism, http://www.who.int/3by5/amds/price/hdd/index.aspx Note: These calculations have been made with the most recent cost published. Drug manufacturers include Cipla (FDC), Bristol-Myers Squibb (stavudine), GlaxoSmithKline (lamivudine), and Boehringer Ingelheim (nevirapine). In all these countries, the annual cost of branded stavudine is US$55 and that of branded lamivudine is US$70; nevirapine accounts for the rest of the cost.

## Conclusion: Prioritizing Health in Patent Law and “Free” Trade Agreements

When it comes to IP law, international trade agreements, and access to ART, the world's poor deserve urgent, honest answers to a number of crucial questions (Box 3). At a time when powerful countries use their financial leverage to negotiate trade agreements that expand their markets—dictating a new global economic order that has far-reaching public health implications—the promotion of global health rests upon a thorough consideration of these questions. Although poverty, inadequate public health infrastructure, lack of political commitment, and poor drug quality certainly contribute to inadequate HIV treatment and are issues with which to contend, international patent law is another structural factor with dire implications for ART in resource-poor settings.

**Box 3. Crucial Questions about IP Law, Trade Agreements, and Public Health**

- Should matters of health constitute a state of exception from patent law?
- What potential benefits do bilateral, regional, and multilateral trade agreements have for resource-poor settings?
- Do current trade agreements respect the national patent law of sovereign states and allow these nations to prioritize public health?
- Will trade agreements stymie efforts to combat global disease, especially the AIDS pandemic?
- Can the IP components of trade agreements be designed in a manner that is mutually beneficial for patients and for drug innovation?

With both the intensification of trade negotiations and concern about the impact of trade liberalization on developing countries, it is vital to formulate alternative strategies that promise to mitigate the impact of strengthened IP law upon poor patients. One such example is the Technological Network on HIV/AIDS, a consortium including Brazil, Cuba, China, Nigeria, Russia, Thailand, and Ukraine, and potentially Uruguay, India, and South Africa, that aims to achieve self-sufficiency in the research, development, production, and distribution of ARVs and other related medications [52,53]. In addition, these countries aim to critically engage IP law in order to ensure that patents do not prevent appropriate care of the sick. Brazil has led these efforts by reforming its laws to be able to break patents and by repeatedly threatening to break patents in order to continue providing free ART for all HIV-positive Brazilians; such threats resulted in dramatic ART price reductions from brand-name pharmaceutical companies [54]. Brazil continues to encourage the disavowal of patents that hinder the provision of health care in low- and middle-income countries [14]. Such courageous efforts must be publicly and financially supported.

Through interdisciplinary efforts, the strengthening of IP law can be effectively challenged in the interests of promoting global health equity. Ultimately, increased research and advocacy must aim to effect concrete changes in the ways that IP provisions are integrated into trade agreements. Such changes require that governments and pharmaceutical companies are held responsible for their self-proclaimed commitments to the common good. There are many avenues for promoting these goals. WHO should have a stronger position in bilateral, regional, and multilateral trade negotiations to ensure that public health remains a priority. In addition, WTO could create a working group on health, as has been suggested [55], whose recommendations would be based on WHO guidelines and recommendations. Low- and middle-income countries could simultaneously agree to restrict IP law discussions to WTO forums, thereby preventing the strong-arming of smaller governments in bilateral, regional, and multilateral trade negotiations. By supporting each other and working within the WTO, smaller countries will occupy a stronger negotiating position in making public health demands. Finally, partnerships such as the Global Alliance for TB Drug Development should be more actively supported to allow for the development of drugs that are free of patent restrictions and address the diseases of the poor.

## Abbreviations

ART: antiretroviral therapy
ARV: antiretroviral
CAFTA: Central American Free Trade Agreement
FTA: free trade agreement
IP: intellectual property
PEPFAR: President's Emergency Plan for AIDS Relief
TRIPS: Trade-Related Aspects of Intellectual Property Rights
WHO: World Health Organization
WTO: World Trade Organization

## References

[pmed-0030332-b001] Schmit J (2005 December 7). Avian flu scare has Tamiflu maker navigating minefield. USA Today. http://www.usatoday.com/money/industries/health/drugs/2005-12-06-roche-tamiflu_x.htm.

[pmed-0030332-b002] United Nations (2005 December 6). Bird flu: Annan seeks affordable drugs for poor states, meets pharmaceutical CEO. UN News Service. http://www.un.org/apps/news/story.asp?NewsID=16824&Cr=bird&Cr1=flu.

[pmed-0030332-b003] Consumer Project on Technology (2001 October 18). Letter from Ralph Nader and James Love to DHHS Secretary Tommy Thompson. http://www.cptech.org/ip/health/cl/cipro/nadethom10182001.html.

[pmed-0030332-b004] World Trade Organization (2001 November 20). Declaration on the TRIPS agreement and public health. http://www.wto.org/english/thewto_e/minist_e/min01_e/mindecl_trips_e.htm.

[pmed-0030332-b005] United Nations (2005). The criteria for the identification of the LCDs. http://www.un.org/special-rep/ohrlls/ldc/ldc%20criteria.htm.

[pmed-0030332-b006] World Trade Organization (1994). TRIPS: Agreement on trade-related aspects of intellectual property rights. Part II—Standards concerning the availability, scope and use of intellectual property rights. http://www.wto.org/english/tratop_e/trips_e/t_agm3c_e.htm.

[pmed-0030332-b007] Loff B (2002). No agreement reached in talks on access to cheap drugs. Lancet.

[pmed-0030332-b008] Khor M (2005 October 26). Impasse on talks on TRIPS and health “permanent solution.” Third World Network. http://www.health-now.org/site/article.php?articleId=499&menuId=1.

[pmed-0030332-b009] Médecins Sans Frontières (2005 October 24). MSF to WTO: Re-think access to lifesaving drugs now. http://www.msf.org/msfinternational/invoke.cfm?objectid=224B1730-E018-0C72-091E8829E29F80E6&component=toolkit.article&method=full_html.

[pmed-0030332-b010] Cage S (2005 December 6). WTO OKs measures to improve drug access.

[pmed-0030332-b011] Pharmaceutical Research and Manufacturers of America [PhRMA] (2005 December 6). PhRMA welcomes TRIPS and public health agreement. http://www.phrma.org/news_room/press_releases/phrma_welcomes_trips_and_public_health_agreement/.

[pmed-0030332-b012] Oliveira MA, Bermudez JAZ, Chaves GC, Velásquez G (2004). Has the implementation of the TRIPS agreement in Latin America and the Caribbean produced intellectual property legislation that favours public health?. Bull World Health Organ.

[pmed-0030332-b013] Adelman K (2005 May 9). Praise for piracy? The Washington Times. http://www.washtimes.com/commentary/20050508-101115-9559r.htm.

[pmed-0030332-b014] (2005 March 16). Brazil requests voluntary licensing for AIDS drugs to treat more patients, reduce costs of importing patented drugs. Kaiser Daily HIV/AIDS Report. http://www.kaisernetwork.org/daily_reports/rep_index.cfm?hint=1&DR_ID=28706.

[pmed-0030332-b015] Consumer Project on Technology (2005). Examples of health-related compulsory licenses. http://www.cptech.org/ip/health/cl/recent-examples.html.

[pmed-0030332-b016] Chien C (2003). Cheap drugs at what price to innovation: Does the compulsory licensing of pharmaceuticals hurt innovation?. Berkeley Technol Law J.

[pmed-0030332-b017] Pécoul B, Chirac P, Trouiller P, Pinel J (1999). Access to essential drugs in developing countries: A lost battle?. JAMA.

[pmed-0030332-b018] Attaran A (2004). How do patents and economic policies affect access to essential medicines in developing countries?. Health Aff.

[pmed-0030332-b019] Attaran A, Gillespie-White L (2001). Do patents for antiretroviral drugs constrain access to AIDS treatment in Africa?. JAMA.

[pmed-0030332-b020] Dumoulin J, Flori YA, Vinard P, Borel T (2003). World market strategies for drugs to fight AIDS.

[pmed-0030332-b021] Lucchini S, Cisse B, Duran S, de Cenival M, Comiti C (2003). Decrease in prices of antiretroviral drugs for developing countries: From political “philanthropy” to regulated markets?.

[pmed-0030332-b022] Rosenberg T (2001 January 28). Look at Brazil. The New York Times. http://www.nytimes.com/library/magazine/home/20010128mag-aids.html.

[pmed-0030332-b023] Stiglitz J (2004 July 24). New trade pacts betray the poorest partners. The New York Times. http://query.nytimes.com/gst/fullpage.html?sec=health&res=9C06EFD7133BF933A25754C0A9629C8B63.

[pmed-0030332-b024] ‘t Hoen E (2002). TRIPS, pharmaceutical patents, and access to essential medicines: A long way from Seattle to Doha. Chic J Int Law.

[pmed-0030332-b025] ‘t Hoen E (2003). TRIPS, pharmaceutical patents and access to essential medicines: Seattle, Doha, and beyond.

[pmed-0030332-b026] Castro A (2005). Adherence to antiretroviral therapy: Merging the clinical and social course of AIDS. PLoS Med.

[pmed-0030332-b027] Coriat B, Dumoulin J, Flori YA, Barnett T, Souteyrand Y (2003). Patents, generic drugs and the markets for antiretrovirals. In: Economics of AIDS and access to HIV/AIDS care in developing countries: Issues and challenges.

[pmed-0030332-b028] Kremer M, Glennerster R (2004). Strong medicine: Creating incentives for pharmaceutical research on neglected diseases.

[pmed-0030332-b029] Joint United Nations Programme on HIV/AIDS [UNAIDS] (2005). http://www.unaids.org/epi/2005/doc/report_pdf.asp.

[pmed-0030332-b030] Farmer P, Léandre F, Mukherjee JS, Claude MS, Nevil P (2001). Community-based approaches to HIV treatment in resource-poor settings. Lancet.

[pmed-0030332-b031] World Health Organization [WHO] (2006). Progress on global access to HIV antiretroviral therapy: A report on “3 by 5” and beyond. http://www.who.int/hiv/fullreport_en_highres.pdf.

[pmed-0030332-b032] World Health Organization [WHO] (2004). The WHO model list of essential medicines: 14th edition. http://www.who.int/medicines/publications/essentialmedicines/en/.

[pmed-0030332-b033] American Foundation for AIDS Research (2004). TREAT Asia special report: Expanded availability of HIV/AIDS drugs in Asia creates urgent need for trained doctors. http://www.amfar.org/cgi-bin/iowa/asia/about/index.html?record=15.

[pmed-0030332-b034] Joint United Nations Programme on HIV/AIDS, World Health Organization (2004). India: Epidemiological fact sheets on HIV and sexually transmitted diseases—2004 update. http://data.unaids.org/Publications/Fact-Sheets01/India_EN.pdf.

[pmed-0030332-b035] Shaw DG (2004 December 27). Statement by Secretary General of the Indian Pharmaceutical Alliance: Strike a balance on product patents—There are perverse incentives in the current system. Discussions of Intellectual Property and Health Care (Ip-health). http://lists.essential.org/pipermail/ip-health/2004-December/007314.html.

[pmed-0030332-b036] Médecins Sans Frontières (2005 March 15). Sources of affordable generic medicines drying up? India should ensure global access to medicines when amending its patent law. New York: Médecins Sans Frontières. http://www.doctorswithoutborders.org/pr/2005/03-15-2005.cfm.

[pmed-0030332-b037] McNeil DG (2005 March 24). India alters law on drug patents. The New York Times. http://www.nytimes.com/2005/03/24/international/asia/24aids.html.

[pmed-0030332-b038] (2005 January 18). India's choice [editorial]. The New York Times. http://www.nytimes.com/2005/01/18/opinion/18tues2.html.

[pmed-0030332-b039] Abbott FM (2005). The WTO medicines decision: World pharmaceutical trade and the protection of public health. Am J Int Law.

[pmed-0030332-b040] Kim J (2004 December 17). WHO Letter to India's Health Minister on Patent Legislation. http://www.cptech.org/ip/health/c/india/who12172004.html.

[pmed-0030332-b041] Joint Action Committee Against Amendment of the Indian Patents Act (2004). Declaration of the joint action committee against amendment of the Indian Patents Act—December 29, 2004. http://www.cptech.org/ip/health/c/india/ngodeclaration12292004.html.

[pmed-0030332-b042] Office of the United States Global AIDS Coordinator (2004). The President's Emergency Plan for AIDS Relief: U.S. five-year global HIV/AIDS strategy. http://www.state.gov/documents/organization/29831.pdf.

[pmed-0030332-b043] Office of the United States Global AIDS Coordinator (2005). Engendering bold leadership: First annual report to Congress on the President's Emergency Plan for AIDS Relief. http://www.state.gov/s/gac/rl/c14961.htm.

[pmed-0030332-b044] The Group of Eight (2005 July 8). Gleneagles summit documents, chair's summary. http://www.g8.gov.uk/servlet/Front?pagename=OpenMarket/Xcelerate/ShowPage&c=Page&cid=1119518698846.

[pmed-0030332-b045] Médecins Sans Frontières (2004). Access to medicines at risk across the globe: What to watch out for in the free trade agreements with the United States. http://www.accessmed-msf.org/documents/ftabriefingenglish.pdf.

[pmed-0030332-b046] Office of the United States Trade Representative (2004). The Central America–Dominican Republic–United States free trade agreement. http://www.ustr.gov/Trade_Agreements/Bilateral/CAFTA/CAFTA-DR_Final_Texts/Section_Index.html.

[pmed-0030332-b047] Khor M (2006 May 24). South American ministers vow to avoid TRIPS-plus measures. Third World Network. http://www.twnside.org.sg/title2/twninfo414.htm.

[pmed-0030332-b048] Russell A (2004 October 9). House Democrats criticize administration over IPR provisions in FTAS. Discussions of Intellectual Property and Health Care (Ip-health). http://lists.essential.org/pipermail/ip-health/2004-October/007018.html.

[pmed-0030332-b049] (2005 January 26). FDA approves generic antiretroviral drug combination, allowing PEPFAR to purchase drugs for use in developing countries. Kaiser Daily HIV/AIDS Report. http://www.kaisernetwork.org/daily_reports/rep_index.cfm?DR_ID=27788.

[pmed-0030332-b050] U.S. Food and Drug Administration (2005 June 20). FDA tentatively approves first generic nevirapine under the President's Emergency Plan for AIDS Relief. FDA News. http://www.fda.gov/bbs/topics/NEWS/2005/NEW01187.html.

[pmed-0030332-b051] U.S. Food and Drug Administration (2005 July 8). HHS/FDA tentatively approves another first-time generic AIDS drug associated with the President's Emergency Plan for AIDS Relief. FDA News. http://www.fda.gov/bbs/topics/NEWS/2005/NEW01202.html.

[pmed-0030332-b052] Morel CM, Acharya T, Broun D, Dangi A, Elias C (2005). Health innovation networks to help developing countries address neglected diseases. Science.

[pmed-0030332-b053] World Health Organization [WHO] (2005). Progress on global access to HIV antiretroviral therapy: An update on “3 by 5”. http://www.who.int/3by5/fullreportJune2005.pdf.

[pmed-0030332-b054] (2003 November 18). New anti-HIV drug deal for Brazil. BBC News. http://news.bbc.co.uk/1/hi/world/americas/3281683.stm.

[pmed-0030332-b055] Kimball AM (2006). The health of nations: Happy birthday WTO. Lancet.

